# Trust by Proxy

**DOI:** 10.7759/cureus.49130

**Published:** 2023-11-20

**Authors:** Mehr Grewal, Eric J Chow

**Affiliations:** 1 Post-COVID Recovery and Rehabilitation Research Group, University of Washington, Seattle, USA; 2 Department of Public Health, Public Health - Seattle and King County, Seattle, USA; 3 Division of Allergy and Infectious Diseases, Department of Medicine, University of Washington, Seattle, USA; 4 Department of Epidemiology, University of Washington, Seattle, USA

**Keywords:** covid-19, community engagement, trust in physician, misinformation, public health messaging

## Abstract

The coronavirus disease 2019* *(COVID-19) pandemic revealed the importance of improving the accessibility of quality public health messaging especially among underserved communities. By establishing a robust communication infrastructure, communities may begin to address the disparities exacerbated by misinformation. In this article, we describe the work done by Worth a Shot, a community-led organization that partnered with trusted, informed county residents; the county residents served as "public health ambassadors" who provided accurate, timely information to their communities in a culturally sensitive approach. This youth-led work may serve as a model for other communities that seek to improve outreach to underserved communities.

## Editorial

“Look around you. I’m not sick, you’re not sick, nobody is sick. I repeat, there is no COVID (coronavirus disease) in these premises,” the gentleman emphasized, gesturing to the church and community center where we were gathered. It was March 2020, and I was there with a group of volunteers, distributing hand sanitizers and masks in light of the usual Sunday congregation. While the world was grappling with the declaration of emergency of international concern, my heart sank as I heard his words repeating in my mind. This was my community, one that I cared deeply about. And this gentleman was a trusted community leader whose family I was close with; his grandson was a high school classmate of mine. To hear him amplify misinformation while many around me struggled to decipher the thicket of COVID-19 news left me confused and discouraged. This type of inaccurate information led many in the community to let their guard down, leading to infection, hospitalization, and sometimes death that may otherwise have been preventable.

In December 2020, news of highly efficacious COVID 2019 (COVID-19) vaccines was announced [1], marking a significant turning point in the public health pandemic response. Anticipating initial vaccine hesitancy [2], I jumped at the opportunity to rally my community. Early on, these efforts included bringing together community physicians, college students, and medical students to develop data-driven education material to inform the community of the benefits of vaccines. Being mindful of the diverse population in our community, informational packets were brought to local community hubs in 26 different languages. We distributed these to more than 200 churches, community centers, and grocery stores across the state, not only in the Seattle area but also in the more rural areas of eastern Washington. Our activities were frequently paired with local public health department outreach to deliver vaccine messaging and vaccines to underserved communities. Our group trained 512 speakers, 449 community advocates, and 256 health leads, and held 23 information sessions. They, in turn, informed their own communities about the importance of vaccine uptake. These combined efforts ultimately coalesced into a nonprofit organization, Worth A Shot [3], with a mission to amplify public health messaging through community advocacy.

During the early days of our organization, activities were centered on bringing public health resources and messaging to communities hit hardest by the pandemic. We set up Seattle Street Sinks [4] at business locations around the greater Seattle area to encourage hand hygiene as the first line of defense against the spread of COVID-19 and other illnesses. We partnered with a community hospital to distribute hand sanitizers, masks, sanitizing wipes, thermometers, and other health essentials to the community during our biannual health fairs. Our most successful activity was our community ambassador program involving more than 300 medical students and healthcare professionals, many of them immigrants and refugees who spoke a variety of languages and served as community ambassadors to raise awareness and answer questions from their communities. Through our program, medical students were able to develop and enhance their professional identity, which helped to reaffirm their commitment to public health. Our activities evolved with the pandemic, initially focusing on preventive measure messaging and then broadening to include COVID-19 vaccination and informing the community.

As our organization's members grew, so did our scope of work. We began locally in the Seattle metropolitan area, but gradually expanded to other parts of Washington State, the Pacific Northwest, and then to different regions of the United States (US). As of 2023, we also have international chapters located in Germany, India, Haiti, the United Kingdom (UK), and Nigeria. It was becoming clear to me that it was the young people in the community who were eager to support our work. It was these youth-driven activities that were truly having an impact on community conversations. Through our activities, we would strive towards a mission to educate and empower youth to be health advocates and public health leaders in their own communities. As a direct result, Worth a Shot was loosening the grip of misinformation while also training the next generation of community leaders, scientists, and entrepreneurs. I initially assumed that the body of work we would embark on would require medical degrees and years of training that only individuals established in their careers would offer. But in our youth sessions, we talked about how community youth could make a difference by influencing the conversations and narratives in their communities in a powerful way. By educating and empowering young people, we were also reaching their parents and family members, communicating in a way that their families understood.

I sought to extend a youth seminar invitation to my classmate whose grandfather inspired me to take those first steps months ago. He seemed intrigued but also hesitant. “My family keeps saying that all these stories about the virus are made up. I’m not sure whom to believe.” I shared that the session would include expert physicians from the community to answer any questions he would have and bring clarity to the topics he had on his mind. Not only was he able to attend our session, but he also asked many questions that reflected general community sentiments and perceptions. The questions were reflective of the distrust and skepticism that had been pervasive in the community.

“I heard that the vaccine is an mRNA vaccine,” he asked. “Wouldn’t it alter my DNA?”

The allergy/immunology physician we had on the panel provided reassurance that the mRNA from the vaccine would not enter the nucleus of the cell, where the DNA is stored. Moreover, the mRNA would degrade after the necessary proteins were made.

“These vaccines were developed so fast!” he exclaimed. “How could we possibly trust them to be safe?”

This time it was the infectious disease physician’s turn to answer. She was familiar with the process and had collaborated closely with people involved in the policymaking.

“Normally, it takes years to design, test, and authorize a vaccine,” she said. “But in light of the current public health emergency, billions of dollars were given worldwide for the vaccine development, which allowed its developers to make rapid progress. Normally, companies may have to wait years to get funding for their work. They underwent rigorous testing, with just as much scrutiny as would normally be given.” 

Behind each of the questions was someone who was sifting through the multiple data sources to get to the truth. I followed up with him after the session and discovered that, in fact, language was a barrier to accessing accurate vaccine information, and frequently educational material was not available to his parents. He overcame this obstacle and shared the information he learned that night. Several weeks later I heard a familiar voice while volunteering at my church’s community center.

“Hi, everyone,” he said a little hesitantly. “I made some comments about COVID-19 when I didn’t want to believe what everyone was saying. Recently, someone shared the truth about COVID-19 with me. I realized I had been wrong. COVID-19 is real. The virus is spreading around our community, and we are at risk because many of us aren’t wearing masks or taking measures to protect ourselves.” He went on to promote mitigation measures including the use of face masks, social distancing, and the uptake of vaccines. The moment led to a palpable wave of emotion that gripped the community center. I stood at the back, beaming because of what I had just witnessed.

Listening to those words reaffirmed Worth a Shot’s mission to empower the youth of our communities as public health advocates. Whether they realized it or not, these youth leaders were shifting the mindsets of the community in the face of an onslaught of misinformation that is now pervasive in our society. The youth were more knowledgeable and experienced than society gave them credit for. They were able to nimbly navigate the nuances of social media platforms, bridging the information gap in the conversation with their families. These changes could begin with a simple remark at the dinner table. More than ever, children of immigrants or refugees help parents and other family members navigate the informational world around them. By reaching young people today, we were making an impact here and now.

While the impact of our programs has been palpable through the >10,000 youth and families we have connected with worldwide, I am most inspired by the experience shared by youth who were able to shape community conversations because of the activities we have sponsored. One such story came from a girl from France whose parents were both immigrants and faced language barriers. Because they had not been able to access public health messaging, they remained unvaccinated. However, after a Worth a Shot seminar, she was able to explain the science behind the vaccine and helped both her parents get vaccinated a month later.

These stories are daily reminders that the impact and influence of youth on their communities should not be undervalued [5]. They shape procedures and policies and shift the mindsets of communities, all behind the scenes. Improving our societies doesn’t have to involve elaborate community programs. In fact, it can start at home, in our local communities. When you witness these community-level changes started by determined youth, you know the investment was worth a shot.
